# Coagulopathy and thromboembolic events a pathogenic mechanism of COVID‐19 associated with mortality: An updated review

**DOI:** 10.1002/jcla.24941

**Published:** 2023-07-11

**Authors:** Parastoo Yousefi, Saber Soltani, Goli Siri, Sara Akhavan Rezayat, Ali Gholami, Alireza Zafarani, Mohamad Hossein Razizadeh, Ehsan Alborzi, Golnaz Mokhtary‐Irani, Behnam Abedi, Sajad Karampoor, Alireza Tabibzadeh, Abbas Farahani

**Affiliations:** ^1^ Department of Virology, School of Medicine Iran University of Medical Sciences Tehran Iran; ^2^ Department of Virology, School of Public Health Tehran University of Medical Sciences Tehran Iran; ^3^ Department of Internal Medicine, Amir Alam Hospital Tehran University of Medical Sciences Tehran Iran; ^4^ Department of Health Care Management and Economics, School of Public Health Tehran University of Medical Sciences Tehran Iran; ^5^ School of Medicine Arak University of Medical Sciences Arak Iran; ^6^ Department of Hematology and Blood Banking, Faculty of Allied Medicine Iran University of Medical Sciences Tehran Iran; ^7^ Department of Virology, Faculty of Medicine Ahvaz Jondishapur University of Medical Sciences Ahvaz Iran; ^8^ Department of Medical Laboratory Sciences Khomein University of Medical Sciences Khomein Iran; ^9^ Gastrointestinal and Liver Diseases Research Center Iran University of Medical Sciences Tehran Iran; ^10^ Molecular and Medicine Research Center Khomein University of Medical Sciences Khomein Iran

**Keywords:** anticoagulant, blood coagulation, coagulation disorders, coagulopathy, COVID‐19, SARS‐CoV‐2

## Abstract

During 2019, the SARS‐CoV‐2 emerged from China, and during months, COVID‐19 spread in many countries around the world. The expanding data about pathogenesis of this virus could elucidate the exact mechanism by which COVID‐19 caused death in humans. One of the pathogenic mechanisms of this disease is coagulation. Coagulation disorders that affect both venous and arterial systems occur in patients with COVID‐19. The possible mechanism involved in the coagulation could be excessive inflammation induced by SARS‐CoV‐2. However, it is not yet clear well how SARS‐CoV‐2 promotes coagulopathy. However, some factors, such as pulmonary endothelial cell damage and some anticoagulant system disorders, are assumed to have an important role. In this study, we assessed conducted studies about COVID‐19‐induced coagulopathy to obtain clearer vision of the wide range of manifestations and possible pathogenesis mechanisms.

## Graphical abstract

In December 2019, a new coronavirus which named SARS‐CoV‐2 (Severe Acute Respiratory Syndrome Coronavirus‐2) emerged from China, and during months, COVID‐19 spread in many countries around the world. Clinical manifestation spectrum associated with COVID‐19 is various and not completely clear. The disease presentations can range from an asymptomatic infection or mild illness to severe pneumonia, sepsis, loss of organs function, and even death. Although most people with COVID‐19 will face mild manifestations, some patients will demonstrate severe clinical signs that lead to hospitalization and intensive care unit (ICU) admission. These clinical complications include severe lung dysfunction, sepsis, shock, or multiple organ failure. Coagulation disorders and thrombotic complications were reported in COVID‐19 patients. The mechanism of Coagulation disorders is not precise in COVID‐19 patients. The current study tried to provide a comprehensive view of the pathophysiology, mechanisms, therapeutic approaches, and considerations about the coagulopathy during COVID‐19.

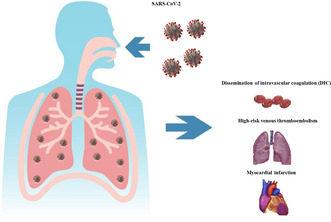

## 1 INTRODUCTION

In December 2019, a new coronavirus which named SARS‐CoV‐2 (Severe Acute Respiratory Syndrome Coronavirus‐2) emerged in Wuhan, Hubei province, China. The conducted disease by this virus is coronavirus disease 2019 (COVID‐19) rapidly spread in many countries around the world.^1^, ^2^, ^3^, ^4^ After a while, this novel coronavirus caused one of the most important health issues worldwide.^5^, ^6^, ^7^, ^8^ Clinical manifestation spectrum associated with COVID‐19 is various and not completely clear. The disease presentations can range from an asymptomatic infection or mild illness to severe pneumonia, sepsis, loss of organs function and even death.^9^, ^10^, ^11^, ^12^ Previous important coronaviruses, SARS‐CoV (SARS‐CoV‐1) and the Middle East respiratory syndrome (MERS‐CoV), responsible for the outbreak in 2002–2003 and 2012, respectively, and associated with severe and fatal diseases.^13^, ^14^, ^15^ Since the start of the pandemic by COVID‐19, different therapeutic approaches are suggested.^16^, ^17^, ^18^, ^19^, ^20^, ^21^, ^22^ Although most people with COVID‐19 will face mild manifestations, some patients will demonstrate severe clinical signs that lead to hospitalization and intensive care unit (ICU) admission. These clinical complications include severe lung dysfunction, sepsis, shock or multiple organ failure.^23^, ^24^ There is a wide range of literature about the severity of COVID‐19 and its potential life‐threatening clinical conditions.^25^ Coagulation disorders and thrombotic complications were reported in the COVID‐19 patients.^26^, ^27^ The mechanism of coagulation disorders is not precise in the COVID‐19 patients. In this regard, there are valuable researches available.^28^ Furthermore, in this study, we conducted studies in COVID‐19‐associated coagulopathy to obtain a clearer vision of this wide range of manifestations and possible pathogenesis mechanisms. In addition, we tried to review the current literature in this particular field to provide an update for clinicians and researchers.

## 2 POSSIBLE MECHANISMS FOR COAGULATION DISORDERS IN COVID‐19

Disorders in coagulation system such as myocardial infarction and thrombotic complications that affect both venous and arterial systems occur in COVID‐19.^29^, ^30^ The coagulopathy conditions of COVID‐19 are not only limited to the primary course of the disease and could be during post‐infection or long COVID‐19 conditions.^31^ These coagulation disorders are due to excessive induction of inflammation, disturbing gas exchange, immobilization and dissemination of intravascular coagulation (DIC).^26^, ^27^, ^32^ It is not yet completely clear how SARS‐CoV‐2 promotes coagulopathy. However, some factors, such as damage to pulmonary endothelial cells and some disorders in the fibrinolysis system, are assumed to have an important role. It is demonstrated that the level of factor VIII, fibrinogen, and also von Willebrand (VWF) in the COVID‐19 disease are higher than in normal cases. Considering this, there is a growing body of research reports, which shows coagulation disorders despite the use of anticoagulant agents.^32^, ^33^, ^34^, ^35^ Some laboratory findings can support a poor prognosis in a patient infected by a novel coronavirus. Among those, some data obtained from coagulation parameters indicate that elevated levels of D‐dimer (>3000 ng/mL) can be considered to predict unfavorable outcomes include death in severe COVID‐19.^1^, ^9^, ^36^, ^37^, ^38^, ^39^ Besides arterial and venous thrombosis, pro‐inflammatory cytokines, which lead to cytokine storm (with unknown causes), are also associated with higher mortality risk.^40^, ^41^ The relationship between these two processes, thrombosis and inflammation, which strengthen each other, has been shown before and is compatible with the above findings.^42^, ^43^ It has been shown that anticoagulant factors like heparin and platelets, besides their original function, also act as anti‐inflammatory and can modulate immune response.^44^, ^45^ Additionally, medications like Dipyridamole with antiplatelet, antiviral and antioxidant features propose to be used in the COVID‐19 hospitalized patients.^46^, ^47^ Although, some patients are still developed thrombosis despite using drugs as a coagulant prevention strategy. Further studies are required due to incomplete documents about heparin efficacy, frequency of a dose and timely administration of anticoagulant agents.^48^ Different studies recently suggested that low‐molecular‐weight heparin (LMWH) would induce statistically significant decrease in mortality rate of severe disease and who present sepsis‐induced coagulopathy (SIC) score ≥4 or D‐dimer levels >6‐fold upper from normal rang.^49^ Disturb coagulation function, including elevated fibrinogen degradation products, factor VIII (FVIII), VWF levels and fibrinogen levels, reported in th COVID‐19 patients.^50^ Derangement of hemostasis associated with proteins involved in the coagulation process can be caused by cytokine storm which leads to a fundamental change in pro and anticoagulant proteins activity.^41^, ^51^ In one study, Christophe et al. report that reactivation of the immune system stimulates coagulation disorders. Moreover, increasing fibrinogen and FVIII levels and disturbing the fibrinolysis process have been shown in all patients infected by COVID‐19.^52^ Disorderliness in other thrombosis and fibrinolysis factors are also associated with severe thrombotic involvement. Levels of tPA (tissue‐type plasminogen activator), TAFIa/I (thrombin activatable fibrinolysis inhibitor) and PAI‐1 (plasminogen activator inhibitor 1) have a remarkable increase in the number of the critical patient than those patients who did not experience a severe form disease. High levels of TAFI antigen and PAI‐1 seems to be associated with mortality in cardiovascular disease and arterial thrombosis.^32^

## 3 IMMUNE SYSTEM AND COAGULATION

Initiating immune response to invasive viral pathogens leads to disturbing the harmony between factors involved in the homeostatic mechanism associated with coagulation. Regardless of forming a clot, platelets mediate interaction between TLRs (toll‐like receptors) and activate inflammatory cascade to eliminate viral infections.^32^ There are some pieces of evidence about the importance of TLRs in the COVID‐19 pathogenesis and induction of coagulopathy effects.^53^, ^54^, ^55^ Too much‐activated platelet and the cascade of events leads to coagulation blood, responsible for elevated D‐dimer and thrombocytopenia. Apart from these, damages of the vascular endothelium, immune deregulation, increasing von Willebrand factor, and accumulation of macrophage, monocyte, platelet and lymphocyte have significant roles in coagulopathy after viral infection.^32^, ^56^, ^57^, ^58^ There is a relationship between endothelial cell activation and increasing in VWF level. Some observations suggest the elevation of VWF in COVID‐19.^50^, ^59^, ^60^ ADAMTS13 (a disintegrin and metalloproteinase with thrombospondin type 1 motif, member 13) Enzyme, or VWFCP (von Willebrand factor‐cleaving protease), regulates the VWF multimers size during synthesized by endothelial cells and megakaryocytes. A low level of ADAMTS13 can cause thrombotic thrombocytopenic purpura (TTP).^61^, ^62^, ^63^, ^64^ One study reported that ADAMTS13 and ADAMTS13/VFW: Ag ratio among survivors and non‐survivors patients indicated a remarkable decrease in the deceased patients than survivors.^35^

Also, poor clinical prognosis in the COVID‐19 patients with coagulation and death is frequently occurs in patients representing myocardial infarction than in patients without cardiac involvement.^65^ Additionally, level of Troponin‐T in the COVID‐19 patients shows statistically significant association with increase PT, PTT and D‐dimer elevation.

## 4 COAGULATION ABNORMALITIES IN OTHER EMERGING CORONAVIRUSES

Similar to the COVID‐19 infection, coagulation abnormalities, PT and PTT prolongation elevated D‐dimer and high‐risk venous thromboembolism are reported in SARS‐CoV.^32^, ^66^, ^67^ However, finding from lung autopsies in patients with SARS‐CoV typically identified pulmonary thromboembolism.^67^, ^68^ A postmortem autopsies study on a SARS‐CoV‐infected case indicated polyangiitis and blood vessel disorders lead to multi‐organ failure.^32^ A few reported also implicated hypercoagulability in MERS‐CoV with a high incidence of DIC in deceased patients.^3^, ^32^, ^69^

## 5 COVID‐19 COAGULOPATHY AND INDUCED THROMBOEMBOLIC EVENTS

Dysregulation in coagulation and the anti‐thrombotic system lead to thromboembolic events in COVID‐19.^70^, ^71^, ^72^ These thromboembolic events could be represented in a variety of clinical manifestations. There are different terminology and clinical conditions for thromboembolic events. These phenomena could be exhibited as thrombosis, embolism, deep vein thrombosis (DVT) and venus thromboembolism (VTE). Furthermore, a summary of highlighted researches in the area of coagulopathy events and thrombosis are provided in Table 1.

**TABLE 1:** A summary of important studies in the field of the DVT, PE and VTE.

Author name	Country	Population	Age	Background disease	Gender (male/female)	Prevalence	Pharmacological intervention	Main outcome	Ref.
Nahum	French	34	62 ± 8	DBT 15, HTN 13	29/5	23	–	Better prognosis for anticoagulation treatment but despite anticoagulation the DVT should be consider	^78^
Demelo‐Rodrígueza	Spain	156	66.7 ± 15.2	–	102/54	23	Enoxaparin and bemiparin	D‐dimer >1570 ng/mL is associated with asymptomatic DVT	^174^
Giorgi‐Pierfranceschi	Italy	124	71.8	–	77/46	9 and PE 5	Enoxaparin	High prevalence of asymptomatic proximal DVT in hospitalized patients	^79^
Longchamp	Switzerland	25	68	Chronic therapeutic anticoagulation 2	16/9	DVT 6, PE 5, and 3 both	Heparin	Prevalence of VTE is high (32%) among critically ill patients/VTE occurred early in course of the disease	^175^
Santoliquido	Italy	84	67.6	Obesity 14, HTN 44, DBT 20, CVD 11, Cancer 14, Previous VTE 3	61/23	10	Enoxaparin or fondaparinux	DVT may occur among non‐ICU hospitalized patients for despite thromboprophylaxis	^176^
Ren	China	48	70	HTN 19, DBT 13, CVD 11, Cerebrovascular disease 7	26/22	35	LMWH	High incidence of DVT in critically ill patients	^177^
Zhang	China	143	63	–	74/69	66	LMWH	The prevalence of DVT is high and is associated with adverse outcomes in hospitalized patients	^178^
Zhang	China	81	62	HTN, hyperglycemia, CVD, cerebral infarction, cerebral hemorrhage, malignancy	–	55	LMWH	High incidence of DVT and needs for LMWH and ultrasonography	^179^
Chen	China	88	63	HTN, DBT, malignancy, gastric ulcer, thyroid disease, HBV, fatty liver	54/9	88	LMWH	More effective VTE prevention and management strategies may need	^180^
Cho	USA	158	67.4 ± 14.6	DBT, ascites, CVD, HTN, malignancy	85/73	52	UFH and LMWH	D‐dimer<6494 ng/mL may exclude DVT	^181^
Ierardi	Italy	234	61.6	Asthma 10, Cancer 26, HTN 93	70/164	25	Heparin	High rate of DVT found in severe patients and its correlation with respiratory parameters	^182^
Koleilat	USA	135	64	CVD 16, COPD 13, CKD 28, DBT 51, VTE 10, HTN 94	72/63	18	Apixaban, bivalirudin, UF and LMWH	Increased risk of DVT, D‐dimer and fibrinigen are reflection of the risk	^183^
Pizzolo	Italy	43	66	HTN 23, DBT 6, cancer 4, CVD 14	29/14	12	Enoxaparin	Have high prevalence of VTE regardless of a thromboprophylaxis	^184^
Jeune	France	42	64.6	HTN 20, DBT 13, CVD 7, cancer 3	23/19	8	–	High prevalence asymptomatic DVT in the first days of hospitalization may be related to poor prognosis	^185^
Marone	Italy	101	65	Obesity 4, malignancy 2	–	42 DVT and 24 PE	LMWH	VTE can arise regardless of severity of respiratory impairment	^71^
Trimaille	France	289	62 ± 17	–	171/118	49 VTE, 11 DVT, 40 PE	Enoxaparin, fondaparinux, UFH	Relationship between leukocytosis count and VTE	^186^
Artifoni	France	71	64	HTN 29, DBT 14, cancer 4, history of VTE 5	43/28	DVT 15, PE 7	–	Despite thromboprophylaxis, the risk of VTE is non‐ICU inpatients	^187^
Middeldorp	Netherlands	198	61	History of VTE 10, cancer 6	130/68	DVT 26, VTE 39, PE 13	–	VTE risk is high in ICU patients	^188^
Alonso‐Fernandez	Spain	30	64.5	CVD 12, RCD 5	19/11	15	–	More age and D‐dimer>1 μg/mL presented a high prevalence of PE	^80^
Stefely	USA	102	61	–	68/34	DVT or PE 23%		Factor V activity >150 IU/dL frequent in VTE	^84^
Chen	China	25	65	HTN 10 (40%), DBT5 (20%), CVDs 4 (16%), DVT 1 (4%)	15/10	10	LMWH	Importance of high D‐dimer level	^189^
Leonard‐Lorant	France	106	–	–	80/26	32	–	D‐dimer>2660 μg/L had a great sensitivity and specificity for PE	1, 83
Minuz	Italy	10	–	4 HTN, 1 thromboembolic diseases	–	6	–	PE evaluation in high D‐dimer	^190^
Bompard	France	134	64	–	94/40	32	LMWH	Prophylactic anticoagulation did not avoid PE	^191^
Cobelli	Italy	55	62	22 HTN, 7 CVD, 11 DBT, 4 COPD, 1 CKD	39/16	28	–	PE overlapping areas of ground‐glass opacities	^192^
Al‐Samkari	USA	3239	61	464 DBT, 1977 HTN, 273 COPD, 431 CVD, 407 CKD	2088/1141	204	–	Early therapeutic anticoagulation did not affect survival	^193^
Patell	USA	163	–	–	–	4	Enoxaparin, Heparin	High rate of thrombosis after the discharge	^194^
Voicu	France	56	–	26 HTN, 25 DBT, 11 CVD	42/14	20	Enoxaparin, Heparin	DVT patients had significantly higher D‐dimer compared with non‐DVT patients but no difference in fibrinogen	^195^

Abbreviations: CKD, chronic kidney disease; CVD, Cardiovascular; DBT, diabetes; HBV, Hepatitis B virus infection; HTN, Hypertension; LMWH, Low molecular weight heparin; RCD, Respiratory chronic disease; UFH, unfractionated subcutaneous heparin; VTE, Venous thromboembolism.

### 5.1 Venus thromboembolism (VTE)

VTE events, in the form of the DVT, cerebral venous thrombosis (CVT), or pulmonary embolism (PE), are estimated as the cause of death in 5%–10% of hospitalized individuals with SARS‐CoV‐2.^73^, ^74^ VTEs are includes DVT, PE, CVT, or even a combination of these conditions. The incidence of VTE in the COVID‐19 patients was in range of 7%–26% based on the different clinical conditions of patients.^75^ There are a significant number of studies about VTE and COVID‐19. Conducted studies on VTE suggested a higher incidence of the VTE in ICU (Intensive Care Units) admitted patients.^76^ Regardless of other conducted reports in the field of coagulopathy and thromboembolic events in COVID‐19, a conducted study by Mei et al.,^77^ there was no statistically higher incidence of the VTE in comparison of the COVID‐19 hospitalized and community‐acquired pneumonia (CAP). Nevertheless, even Mei et al.^77^ mention COVID‐19‐associated hypercoagulability. Furthermore, elevated D‐dimer was the most frequent laboratory finding. Besides that, Nahum et al.^78^ highlighted a better prognosis for anticoagulation treatment in the COVID‐19 patients and the importance of the DVT consideration in these patients. This importance of the DVT could be an emphasis by considering the Giorgi‐Pierfranceschi and colleagues' study either.^79^ Giorgi‐Pierfranceschi indicates higher prevalence of patients with asymptomatic DVT and associated PE in hospitalized individuals infected by SARS‐CoV‐2.

Alonso‐Fernández and colleagues^80^ concluded an increased age and D‐dimer >1 μg/mL are frequent in COVID‐19 with PE presentation. The importance of D‐dimer monitoring was also suggested by other studies.^81^, ^82^, ^83^ Regardless of the D‐dimer level, the factor V activity is suggested as a biomarker for PE monitoring during COVID‐19 by Stefely et al.^84^

Meanwhile, investigation of cerebral venous sinus thrombosis represents good outcomes in the most COVID‐19 patients with CVT.^85^ In addition, the gender assessment indicates the female gender as the dominant portion of the patients. Furthermore, Cavalcanti and colleagues^86^ study suggested that the median duration between the COVID‐19 onset of symptom to development of thromboembolic events in patients with CVT is 2–7 days.

### 5.2 Anticoagulant treatment

There are varieties of studies investigating the anticoagulant treatment guidelines in the COVID‐19 patients.^87^, ^88^, ^89^, ^90^ Highlighted researches are listed in Table 2. These studies indicate that the Heparin/LMWH is used in the most common anticoagulation strategy, including for a wide range of the coagulopathy events such as DVT and VTE. In addition, heparin's anticoagulant activity is also familiar with antiviral activity against enveloped viruses, including coronaviruses.^91^, ^92^ This property of heparin results from the binding of viral glycoproteins to glycosaminoglycans (GAGS) such as heparan sulfate (HS) that have widespread functions within the body. In herpes simplex virus (HSV) infections, heparin competition with surface glycoproteins in host cells can inhibit viral entrance. In addition, in Zika virus infection heparin can reduce human neural progenitor cell death.^93^, ^94^, ^95^ Moreover, it has indicated that soluble heparin can bind to SARS‐CoV‐2 spike, leading to viral entry inhibition.^96^, ^97^, ^98^, ^99^

**TABLE 2:** Highlighted researches in the field of therapeutic approaches.

First author	Country	Patients number	Pharmacological intervention		Objective	Patients background condition	Maine study outcome	Ref.
			Drug	Dose (mg or UI per day)				
Santoro	Spain, Italy, Ecuador, Cuba, Germany, China, Canada	7824	Single or dual APT	–	APT efficacy in COVID	HTN, DBT, CVD	APT during hospitalisation could be associated with lower mortality, shorter mechanical ventilation and no risk of bleeding	^196^
Spiegelenberg	Netherlands	1154	92 VKA and 98 DOAC	–	Prior therapeutic anticoagulation use and clinical outcome	–	No associations between prior use of therapeutic anticoagulation and overall mortality	^197^
Tremblay	USA	3772	Anticoagulant or APT	–	Empiric therapeutic anticoagulation	Lung disease, history of VTE, obesity	AC alone is unlikely to be protective for COVID‐19–related morbidity and mortality	^198^
Rivera‐Caravaca	HOPE registery	110	VKA and DOAC	–	Prior anticoagulant efficacy	CVD	Patients on OAC therapy at hospital admission showed lower survival and higher mortality risk but bias was reported in the comorbidity	^199^
Lachant	USA	107	DOAC, Warfarin, LMWH	–	Chronically anticoagulated before COVID	HTN, DBT, CKD, VTE, COPD	May protect against thrombotic complications and decrease disease severity	^200^
Santoliquido	Italy	84	Enoxaparin or fondaparinux	–	Thromboprophylaxis	HTN, DBT, cancer, VTE	DVT occur among non‐ICU patients despite guideline recommended thromboprophylaxis	^176^
Vlot	Netherlands	16	Anti Xa	0.1–0.3 IU/mL	Anti Xa assessment	HTN	Anti Xa dose adjustment	^201^
			LMWH	7600 IU				
Ayerbe	Spain	2075	Heparin	–	Heparin assessment	Non	14% mortality in Heparin admitted group	^202^
Brouns	Netherlands	101	OAT	–	OAT assessment		No protection against mortality by OAT	^203^
Christie	Georgia	5	tPA	50 mg IV (25 mg bolus)	Case		All five patients improved	^204^
Ferguson	USA	141	LMWH	1.5 mg/kg	DVT prophylaxis		LMWH not useful for DVT prophylaxis in COVID‐19 patients	^205^
Hanif	USA	921	Warfarin, UFH	–	Treatment and prophylaxis for VTE and thrombosis	33 patients with anticoagulant prior to the admission	Patients on therapeutic anticoagulation had a higher mortality compared with patients on prophylactic anticoagulation	^206^
Shi	China	42 (21 case and 21 control)	LMWH	–	LMWH efficacy	Non	LMWH could significantly decrease IL‐6 and improve the Lymphocyte count in COVID‐19 patients	^207^
Sivaloganathan	UK	60 case/120 control	Anticoagulant or APT	–		Non	Pre‐admission anticoagulants or antiplatelet agents did not make difference in COVID‐19 mortality or ICU admission	^208^
Stessel	Belgium	78	LMWH	–		HTN, DBT, CKD, CLD	CRRT could be reduced by implementation of thromboprophylaxis	^209^
Yormaz	Turkey	96 (48 case and 48 control	LMWH	4000 UI	LMWH clinical outcome	HTN, DBT, CAD	LMWH shows improve in laboratory findings, lymphocyte count, CRP, and D‐dimer results	^210^
Mattioli	Italy	105	Enoxaparin	80 mg		HTN, DBT, Obesity, Smoking	Intermediate doses of LMWH could be useful and also safe in patients with CKD	^211^
Rossi	Italy	70	OAT	–	Direct oral anticoagulants	HTN, obesity, DBT	DOAC intake is associated with a decreased mortality risk	^212^
Tang	China	449	LMWH/UFH	–	Heparin efficacy on COVID‐19	HTN, DBT	LMWH associated with better prognosis in severe COVID‐19 patients	^49^

Abbreviations: anti Xa, anti‐factor Xa; APT, antiplatelet therapy; CAD, Coronary Artery Disease; CKD, Chronic kidney disease; CLD, Chronic liver disease; CRRT, continuous renal replacement therapy; DBT, Diabetes; DOAC, direct oral anticoagulants; HTN, hypertension; Non, represents no relevant medical history; OAT, oral antithrombotic therapy; tPA, tissue plasminogen activator; UFH, unfractionated heparin; VKA, vitamin K antagonists.

Despite Heparin's widespread use as an anticoagulant agent, it demonstrated that heparin is associated with anti‐inflammatory activity via interaction and modulates inflammation mediators. This anti‐inflammatory activity leads to a decreased level of IL‐8 and IL‐6.^99^, ^100^, ^101^ heparin can also bind to acute‐phase proteins and complement factors ti induce the anti‐inflammatory function of heparin.^95^, ^101^ In this regard, the importance of anticoagulant therapy for reducing the cytokine storm has been declared.^102^

Using heparin in the COVID‐19 patients will help to decrease the risk of thromboembolic incidence, which may be associated with fatal outcomes. However, the management of bleeding risk and administration of effective doses of drugs should be considered. Close monitoring of the D‐dimer, platelet count and PT (prothrombin time) in all patients with suspected or confirmed COVID‐19 are also recommended. Further investigation for the anti‐inflammatory effect of heparin due to the bidirectional interaction between these two systems, inflammation and coagulation seems to be necessary.

Despite all of the mentioned features and potential benefits of Heparin therapy, there are some concerns. For example, we mentioned Heparin‐induced thrombocytopenia (HIT) (due to the heparin attachment into the platelet factor 4) and Heparin resistance (due to the high‐dose UFH, for instance, UFH > 35,000 IU per day). Although Heparin resistance and HIT are rare events, even in COVID‐19. In the conducted study by Huang et al.,^103^ it's been suggested that heparin therapy and identification of HIT may be an essential element in the COVID‐19 mortality reduction. There is no exact information about the HIT in cases with COVID‐19. However, by considering these limited studies, the anticoagulant Argatroban seems to be a great replacement for fracture heparin in confirmed coronavirus patients involved with thromboembolic events and HIT.

Investigation of the etiologic factors for Heparin resistance in four cases of COVID‐19 suggested the Factor VIII level is increased in patients.^104^ An increase in factor VIII could be a possible pathophysiology for heparin resistance in COVID‐19. Also, a high incidence of heparin resistance was reported by White et al.^105^ This manner highlights the importance of further research in anticoagulation and optimization of the therapeutic dose for the treatment of coagulopathy events or even for prophylaxis in the COVID‐19 patients.

Furthermore, in the context of the anticoagulant treatment for COVID‐19 some points need to be considered include, there is any robust evidence that leads to indicate the unrestricted use of anticoagulants in patients infected with the COVID‐19, without clear confirmation of a thromboembolic event or hospitalization in intensive care.^106^, ^107^ It could be concluded that confirmation of COVID‐19 is not indicative of the use of anticoagulants. Patients in moderate stage of COVID‐19 could be concidered as a candidate for thromboprophylaxis if there was low‐bleeding risk, represents high D‐dimer levels, or require supplemental oxygen.^107^

### 5.3 SARS‐CoV‐2 variants and coagulopathy

Effects of different SARS‐CoV‐2 variants in the severity of disease, epidemiological pattern, and clinical presentation of patients are previously described.^108^ These difference in the severity of disease between variants also affects coagulopathy patterns. There are limited studies about this field of study. The in‐vitro study indicates more potential for coagulation in the alpha variant in compare with the Omicron.^109^ In addition, in clinically evaluated samples, Omicron variants are less competent for micro clot formation or coagulation abnormalities in comparison with alpha, beta and delta variants.^110^, ^111^ As a point of view, it seems that one of the aspects of less sever Omicron variant infection in compare with previous variants is associated with its coagulation alteration profile.

## 6 NOVEL RESEARCH FOR THE PATHOPHYSIOLOGY OF COAGULOPATHY IN COVID‐19

There is some novel pathophysiology for coagulopathy in COVID‐19. In this study, we only provide a view of some of the most important novel mechanisms for coagulopathy in COVID‐19. Some important original researches about Endothelial cell dysfunction, Complement activation, or other important mechanisms are provided in Table 3. More discussion about these mechanisms are provided in the next sections. However, it should be considered that these mechanisms are tight links.

**TABLE 3:** Some highlighted studies about the novel pathogenesis mechanisms of the COVID‐19 associated coagulopathy.

First author	Country	Possible mechanism	Sample		Final conclusion	Ref
			Type	Number		
Cugno	Italy	Complement activation, endothelial damage and disease severity	COVID‐19	148	MAC levels correlated with vWF and paralleled disease severity	^152^
Magro	USA	Complement activation, endothelial damage	COVID‐19	5	Co‐localization Spike with C4d and MAC in lung and skin biopsy, microvascular injury and coagulopathy	^149^
Goshua	USA	Endotheliopathy	COVID‐19	68	Increase in vWF and soluble P‐selectin mortality significantly correlated with VWF antigen and soluble	^59^
Nagashima	Brazil	Endothelial dysfunction	Postmortem	6	Endothelial dysfunction conditions might lead to systemic thrombotic events	^213^
Busch	Netherlands	Hypercoagulability and NETosis	COVID‐19	228	Hypercoagulability and thrombotic events are driven by NETosis, contact activation of coagulation system, and complement	^214^
Cimmino	Italy	IL‐6, Vitamin D and	HUVEC	–	Vitamin D significantly reduced TF, adhesion molecules and ACE2 expression in IL‐6‐treated cells	^215^
Zuo	USA	Hypercoagulability and NETosis	COVID‐19	44	Potential relationship between NETosis and thrombosis	^216^

Abbreviations: CS, Cross‐sectional; HUV, Human Umbilical Vein Endothelial Cells; MAC, membrane attack complex; TF, tissue factor; vWF, von Willebrand factor.

### 6.1 Endothelial cells dysfunction

The vascular endothelium constitutes the inner cellular layer of vessels, which forms a barrier between blood and tissue.^112^ Since it is related to both blood cells and other cells, it plays a critical role in different mechanisms such as maintaining hemostasis, prompting recruitment, adhesion surface and interaction of platelets and leucocytes on thrombogenic surfaces, and regulation of vascular tone through producing various vasoactive substances.^112^, ^113^

The required receptor for attachment of SARS‐CoV‐1 and 2 to epithelial cells is ACE2 which expressed on the surface of these cells. Yang and colleagues showed that epithelial cells infected by SARS‐CoV‐1 could be a target for autoantibodies. Therefore, we can conceive such an outcome in SARS‐CoV‐2.^114^ The infection of endothelial cells with the virus and subsequent effects is the key point to endothelial dysfunction in patients. High levels of pro‐inflammatory cytokines such as IL‐1, IL‐6 and TNF and ferritin activate the endothelial cells of the lung vessels.^115^, ^116^, ^117^ Furthermore, higher levels of VWF were seen in the COVID‐19 patients, which is related to reduced activity of disintegrins metalloproteinase with ADAMTS13.^118^ All these events can eventually lead to the development of thrombotic microangiopathy.^119^ Some other markers that indicate endothelial damage were also investigated in COVID‐19. Goshua and colleagues reported higher levels of P‐selectin (an indicator for endothelial cells and platelet activation) in ICU‐admitted patients. Moreover, they have seen that elevated amounts of thrombomodulin are associated with a higher mortality risk.^59^ The other investigated factor is circulating endothelial cells (CECs), which are detached endothelial cells from damaged vessels. These cells exist in normal individuals at low levels; however, an increased count of CECs denotes the endothelial injury. Since other coronaviruses are cytopathic^120^ and can induce the intracytoplasmic NOD‐like receptor protein 3 (NLRP3) inflammasome, which plays a role in the detachment of endothelial cells, as seen in the case of the Kawasaki disease^121^ and also triggering cytokine storm.^122^ Thereby, CECs are interesting potent markers in studying coagulopathies in COVID‐19. Guervilly and colleagues reported an increased number of CECs in the COVID‐19 patients, especially those who were admitted to ICU. They also showed that CECs are correlated to elevated plasma levels of soluble vascular cell adhesion molecule 1 (sVCAM1).^123^ Falcinelli and colleagues also confirmed these results. In addition to sVCAM1, they reported an increased amount of soluble intercellular adhesion molecule 1 (sICAM1) in COVID‐19.^124^ Taken together, we highly suggest implementing preventing measures such as blocking pro‐inflammatory cytokines and NLRP3 inflammasome as effective measures to reduce the risk of endothelium damage by COVID‐19.

### 6.2 Platelets activation in COVID‐19

Since there is a considerable number of platelets circulating in the blood vessels, they might be the first cells to face the virus in the blood.^125^ The platelet response against the RNA viruses is initiated by the interaction between the viral genome and the toll‐like receptor 7 (TLR7) located in the endolysosomes of platelets.^126^, ^127^ While it is not clear whether platelets express ACE2 or not,^125^ the SARS‐CoV‐2 genome is isolated from platelets by Zaid and colleagues.^128^ The mechanism of internalizing ssRNA viruses and subsequent events have been shown in the case of influenza. The virus can enter the platelets through unknown receptor‐independent mechanisms. Following the entrance of the virus to the platelets and in acidic pH, activation of TLR7 leads to releaseing of α‐granule and interaction P‐selectin and CD40L in platelets with neutrophils.^127^ In addition, activation of TLR7 triggers the release of the complement C3 component. The complement C3 stimulates the phenomena of NETosis by neutrophils, in which they release neutrophil extracellular traps (NETs),^126^ extracellular components consist of cytosolic and granule proteins that are made of decondensed chromatin. NETs traps help the immune system in fighting different pathogens including viruses.^129^ However, they are extremely prothrombotic and induce intravascular coagulation. Furthermore, C3 can also be activated by this thrombin and eventually continue the complement cascade, which plays a major role in inflammation.^130^ Zuo and colleagues have investigated the NET markers expression level such as myeloperoxidase‐DNA complexes, and citrullinated histone H3 in the COVID‐19 patients and compared it with healthy controls. Their results showed that there are higher levels of these markers in the COVID‐19 patients.^131^

The effects of COVID‐19 on platelet activation are comprehensively investigated by Canzano and colleagues. They found that the COVID‐19 patients represent higher levels of tissue factor (TF) positive platelets than healthy individuals and patients with coronary arthritis disease. Also, they found more up‐regulation of P‐selectin and formation of platelet–leukocyte aggregate in these patients. Interestingly, they have observed activation of platelets in healthy individuals' blood after mixture with the plasma of the COVID‐19 patients.^132^, ^133^

### 6.3 Complement activation and coagulopathy in COVID‐19

Innate immunity concidered as the first line of the body's defense against infectious agents. The complement system is a member of innate immune system and is comprised of a course of steps called complement cascade that trigger inflammation against infections and some other events.^134^ The complement can be activated by classic, alternative and lectin pathways. Initiation of all of the mentioned pathways leads to the cleavage of a protein named C3. After cleavage, the protein will be divided into two proteins named C3a, which is a proinflammatory protein, and C3b, which opsonizes the pathogens and also continue the next steps.^135^ C3b mediates the C5 cleavage and release C5a (anaphylatoxin) and, C5b, which is responsible for the formation of a membrane attack complex (C5b‐9).^136^ Albeit the complement plays a major role in the protection of the body, its overreaction can inflict heavy damage to different organs.^136^, ^137^

Besides acting as an anaphylatoxin, C5a increases the tissue factor activity,^138^, ^139^ and plays important role in coagulation.^140^ Furthermore, two proteins that start the lectin pathway, namely MASP1, and MASP2, can activate thrombin by prothrombin cleavage^141^ and also activate fibrinogen and factor XIII.^142^, ^143^ Different studies showed that inhibition of complement cascade resulted in consequences of anticoagulation effects such as prolonged bleeding.^140^, ^144^, ^145^, ^146^

Most of our knowledge about the role of complements in coronavirus infection was obtained from studies on SARS‐CoV‐1 and MERS.^137^ Gralinski and colleagues showed less severe symptoms in C3 deficient mice than intact mice, who were infected with SARS‐CoV‐1.^147^ Jiang and colleagues observed elevated levels of C5a and higher expression of C5a receptor (C5aR) in leukocytes and pneumocytes of MERS‐infected mice. Also, blockage of C5a–C5aR axis lightened the tissue damage in those mice.^148^ In the SARS‐CoV‐2, Magro and colleagues reported deposition of complement fragments including MASP‐2, C5b‐9 and C4d microvasculature of lung tissue of patients with severe symptoms.^149^ According to Gao and colleagues, the N protein on the surface of coronaviruses binds the MASP2 and triggers the complement cascade.^150^ Lam and colleagues used flow cytometry to survey the formation of bonds between red blood cells and some products during complement activation such as C3b, iC3b, C3dg and C4d in hospitalized patients of COVID‐19 compared with healthy individuals and they found a considerable increase in the COVID‐19 patients.^151^ Cugno and colleagues monitored the levels of C5b‐9 and C5a and also VWF in hospitalized the COVID‐19 patients. They observed high levels of these factors in the patients. Moreover, they saw that their concentration decreased during the remission of these patients.^152^ Altogether, this results illustrate the pivotal role of the complement in tissue damage as well as the development of coagulopathies and underlines the importance of preventive measures to control overreaction of this wing of the innate immunity.

### 6.4 Coagulopathy‐induced molecular changes in gene expression

As discussed above, coagulation and fibrinolysis cascade changes cause thrombosis in the COVID‐19 patients that finally leads to poor outcomes or even death. Changes in serum level of coagulation or fibrinolysis cascade in COVID‐19 are known today, so there is an urgent need for evaluating changes in transcriptomic level for better treatment and disease evaluation.

Jha et al. assessed transcriptional profile in lung epithelial cells that infected with different coronaviruses include SARS‐CoV‐2, MERS‐CoV or SARS‐CoV. They showed that changes in transcriptional levels of COVID‐19 infected lung epithelial cells were much different from those of other viruses. Increases F3 gene, which codes tissue factor, and decreases in the TFPI2 gene, which codes TFPI, have been shown in this article.^153^ Jain et al. have investigated nasopharyngeal swaps of the COVID‐19 patients for transcriptomic changes. They found coagulation cascade dysregulated and increased anti‐Fibrinolysis genes, especially SERPINE1 and SERPINF2, which codes PAI‐1 and alpha2AP, respectively.^154^ Mast et al. examined transcriptional profile BALF samples of the COVID‐19 patients. They determined reduction in anti‐coagulant like thrombomodulin and fibrinolysis system like urokinase and its receptor. They found increases in TFPI and no tissue factor increases, so they suggested that other pathways, except FVIIa/TF, can result in thrombosis related to COVID‐19.^155^ Fitzgerald et al. examined circulating immune cells, primary respiratory epithelial cells, patient‐derived bronchial alveolar lavage cells of the COVID‐19 patients. They have found dysregulated coagulation cascade and suppressed plasminogen activation system in, in vitro infected primary respiratory epithelial cells, patients derived bronchial alveolar lavage cells. On the other hand, those changes have not been seen in circulating immune cells. Moreover influenza A infected normal human bronchial epithelial cells infected did not show the same changes with SARS‐CoV‐2.^156^ Gill et al. conducted a study on the leucocyte transcriptional profile between COVID‐19‐associated patients and other ICU‐admitted patients. They showed an increase in expression of VWF, SERPINE1 and GZMB genes in the COVID‐19 positive patients. GZMB, which codes Granzyme B can be involved in thrombosis through damage to endothelial cells.^157^ Manne et al. have performed a study on severe COVID‐19 with hemostatic abnormalities. They have observed an increase in expression level of P‐selectin in COVID‐19. They have also seen an elevation in MAPK pathway and thromboxane activation, leads to platelet hyperactivity related to SARS‐CoV‐2 hypercoagulation.^158^

### 6.5 Renin‐angiotensin‐aldosterone system

Renin‐Angiotensin‐Aldosterone System (RAAS) is a part of the hormone system that spreads throughout of human body. In this system, the angiotensinogen is converted to Ang I through the enzymatic activity of Renin. Then, Ang I is turned to Ang II by angiotensin converting Enzyme (ACE I). Ang II is a stimulator of inflammation and coagulation and its role has been well‐known in cardiovascular disease. ACE II(angiotensin converting Enzyme II) converts Ang II to Ang (1–7), an anti‐inflammatory and anti‐coagulation protein to maintain balance.^159^

Ang II binds to two different receptors named AT1R (angiotensin II type 1 receptor) and AT2R (angiotensin II type 2 receptor). Ang II affinity for AT1R is more than AT2R, and binding this protein to AT1R causes its inflammation and coagulation. Through binding to its receptor, Ang II increases TF and PAI‐1 and improves the chance of shaping thromboses.^160^ So, using RAAS inhibitors, especially angiotensin receptor blockers (ARBs) and ACEIs (angiotensin‐converting enzyme inhibitors), have been discussed in many pathophysiologic situations like COVID‐19.^159^

Kutz et al. have researched RAAS elements in the COVID‐19 patients and showed a decrease in RASS, especially in Ang (1–7), as a protective element.^161^ Because overwhelming inflammatory and hypercoagulation in COVID‐19 using RAAS inhibitors are recommended, there have been some debates about using these drugs, however. Animal studies have shown that these drugs increase ACE II, SARS‐COV‐2 receptor, in the patents, and as already we know, the virus can use it as an entrance to host cells.^162^, ^163^, ^164^ So, many arguments have arisen about continuing or discontinuing RAAS inhibitors after publishing a research letter in the lancet by Fang et al. that mentioned adverse consequences of usage of RAAS inhibitors in the COVID‐19 patients with comorbidities.^165^ Most of the articles have mentioned that using these drugs are beneficial for the COVID‐19 patients, or at least they are neutral. For example, Safizadeh et al. have shown that using RAAS inhibitors in pre‐existing hypertensive the COVID‐19 patients are protective against mortality and hospitalization.^166^ Dublin et al. have shown that, these drugs did not increased risk of mortality and hospitalization.^167^ Loader et al. have stated that the early stages of RAAS inhibitors will not increase the risk of severe outcomes in the COVID‐19 patients,^168^ and research that have been conducted by Park et al. in South Korea showed that taking RAAS inhibitors for the COVID‐19 patients are not harmful.^169^ In addition, some systematic reviews and meta‐analyses have been done, and they have not shown an increased risk of mortality and hospitalization in using RAAS inhibitors.^170^, ^171^, ^172^, ^173^

## 7 CONCLUSION

COVID‐19 research implementation highlights the importance of coagulopathy and thromboembolic events in confirmed coronavirus cases, especially those associated with severe disease and hospitalization. The current study tried to provide a comprehensive view of the pathophysiology, mechanisms, therapeutic approaches and considerations about the coagulopathy during COVID‐19. This recent growing body of knowledge about coagulopathy in COVID‐19 critically needs further studies and could be a lead for coagulopathy disorders in other infectious diseases. High incidence of thrombosis or DVT, PE, during COVID‐19, emphasizes the need to screen coagulation profiles to manage thromboembolic events and optimize the prophylaxis programs for the COVID‐19 hospitalized patients to be required. In addition, we must emphasize the mechanism by which COVID‐19 caused coagulopathy and thromboembolic events, since the elucidation of the involved mechanism in these complications may represent some novel therapeutic avenue for tackling COVID‐19. The underlining mechanisms for coagulopathy in COVID‐19 include complement activation, platelets activation, endothelial cells dysfunction, NETs and RAAS derangement could lead to future studies and possible therapeutic options. It needs to be concidered that, all the mentioned mechanisms needs further investigations for a clear conclusion and possibility to considered as clinical therapeutic option.

## AUTHOR CONTRIBUTIONS

PY, SS, GS, SAR and AF: design and concept of manuscript, AG, AZ, MHR, EA, GMI, BA, SK and AT: data collection and manuscript preparation, SS, PY, AT and AF: manuscript editing and manuscript review, all included authors read and approved the final manuscript.

## CONFLICT OF INTEREST STATEMENT

The authors declare no conflict of interest, financial or otherwise.

## Data Availability

The authors confirm that the data supporting the findings of this study is available within the article.
